# Effects of judo-specific intermittent training on lower-limb impulse and specific performance in judokas

**DOI:** 10.7717/peerj.19491

**Published:** 2025-06-04

**Authors:** Pi-Yen Ho, Hung-Chih Yeh, Fang Li, Chia-An Ho, Cheng-Pang Yang, Ying-Chen Kuo, Chih-Wen Hsu, Chin-Shan Ho

**Affiliations:** 1 Department of Sports Training Science-Combats, National Taiwan Sport University, Taoyuan, Taiwan; 2 Graduate Institute of Sports Science, National Taiwan Sport University, Taoyuan, Taiwan; 3 School of Physical Education, Central China Normal University, Wuhan, China

**Keywords:** Sparring partners, Uchikomi, Ippon Seoi-nage, Mae-ukemi, Special judo fitness test

## Abstract

Intermittent training (IT) has been shown to enhance athletic performance by improving aerobic and anaerobic capacities, neuromuscular efficiency, and recovery key factors for judokas given the intermittent nature of judo combat. This study investigated the effects of a six-week judo-specific intermittent training program on body composition (body mass (BM); percent body fat (PBF); skeletal muscle mass (SMM)), grip strength, lower-limb impulse (countermovement jump, (CMJ)) (relative force peak (RFP)), relative force peak (RFP), reactive strength index-modified (RSImod), jump height (JH), time to takeoff (TTT)), and specialized performance (special judo fitness test, (SJFT)) (SJFT series A, SJFT series B, SJFT series C, SJFT total number of throws, SJFT post-exercise heart rate, SJFT one-minute post-exercise heart rate), with two primary objectives: (1) incorporating uchikomi + ippon seoi-nage (Tori group) as part of the training intervention and (2) examining the impact of serving as sparring partners through mae-ukemi (Uke group) on performance outcomes. Thirty male judo athletes (all black belt) were randomly assigned to the Tori (169.90 ± 6.17 cm in stature, 76.67 ± 18.2 kg in mass, 20.64 ± 3.07 years of age and 13.10 ± 2.88 years of experience), Uke (72.36 ± 6.32 cm in stature, 77.28 ± 19.4 kg in mass, 21.73 ± 6.15 years of age and 11.90 ± 1.79 years of experience), and control group (CON) (167.18 ± 4.16 cm in stature, 76.77 ± 13.7 kg in mass, 21.55 ± 5.30 years of age, and 12.00 ± 2.40 years of experience) (*n* = 10 per group). The intermittent training sessions were held twice weekly (Tuesday and Thursday) at 2:00 PM, with a 24-hour recovery between sessions. Participants were paired by body weight (≤10% difference). Training included two blocks of six 20-second sets, with 20-second passive rest intervals, totaling 8 minutes per session. The results showed a significant time effect (*p* < 0.01) in the special judo fitness index, along with a time × group interaction (*F* = 5.44; *p* = 0.01; *η*^2^ = 0.28). *Post hoc* comparisons revealed that the Tori group’s improvement was significantly greater than that of the other two groups (Tori: *p* < 0.01; Uke: *p* = 0.58; CON: *p* = 0.78). For CMJ parameters (RFP, JH, RSImod), although a significant time effect was observed, no interaction effects were found for any parameter. In terms of body composition and grip strength, neither a time effect nor an interaction effect was present. Additionally, the Uke group, while serving as sparring partners, had no negative impact on any variables examined in this study. Judo-specific intermittent training can significantly enhance SJFT, making it an effective training method. While CMJ parameters showed a time effect, intermittent training alone may not fully improve lower-limb impulse. Additionally, during sport-specific intermittent training, serving as a sparring partner does not negatively impact performance, allowing safe integration into training programs.

## Introduction

Judo is a highly intense combat sport that involves various motor skills, such as throws and grips, and demands diverse athletic abilities, such as velocity, strength, and agility. The ability to repeatedly perform high-intensity intervals, enabling judokas to gain an advantage by knocking down opponents or securing holds on the floor, has become essential for achieving excellence in judo (Franchini et al., 2011; Franco-Alvarenga et al., 2019). Performance in judo competitions depends on technical and tactical developments that require a high level of physical fitness to cope with the intensity of the competition and maintain the technical quality of judo techniques throughout the fight (Miarka et al., 2012). However, these competitions, interspersed throughout the year, often demand high-intensity training, recovery, or tapering for athletes to achieve their best performance during the competition (Franchini et al., 2016). Moreover, during the competition period, athletes may be required to participate in five to seven contests in a single day with their maximum effort exerted in each match. This not only highlights the highly intense and demanding nature of judo competitions but also provides strong evidence for the crucial role of excellent physical fitness and recovery ability in achieving optimal combat performance.

The Special Judo Fitness Test (SJFT) assesses judo performance by simulating match conditions. Athletes move between two partners six meters apart while continuously executing ippon-seoi-nage, a key judo technique (Choi & Song, 2023). The test evaluates anaerobic impulse, aerobic endurance, and recovery ability, with key indicators including total number of throws, heart rate immediately after the test, heart rate one minute after, and the SJFT index. The test evaluates anaerobic capacity, aerobic endurance, and recovery capacity, with key indicators including the total number of throws, heart rate immediately after the test, heart rate one minute after the test, and the SJFT index. A higher number of throws indicates greater anaerobic capacity, a lower post-test heart rate reflects better aerobic endurance, and a faster heart rate reduction indicates superior recovery capacity (Agostinho et al., 2018; Campos et al., 2022; Kons et al., 2021; Paulo Lopes-Silva et al., 2021; Sterkowicz-Przybycień, Fukuda & Franchini, 2019). The countermovement jump (CMJ) is a crucial indicator of lower limb impulse and neuromuscular efficiency in judo (Kons et al., 2023). Higher CMJ performance is closely linked to greater throwing efficiency, quicker counterattacks, and sustained impulse during matches. Since judo requires repeated high-intensity movements, athletes with strong CMJ capabilities can minimize fatigue effects and maintain optimal performance (Chang et al., 2022). This is largely due to the stretch-shortening cycle (SSC), which enhances impulse output by efficiently utilizing stored elastic energy during actions. In judo, SSC plays a key role in generating forceful throws and swift footwork, making improvements in CMJ and SSC function essential for maximizing performance (Kons et al., 2023). Detanico et al. (2012) confirmed a significant correlation between CMJ performance and ippon seoi-nage, with a high correlation observed (*r* = 0.74; *p* < 0.01). Therefore, this study aims to investigate whether sport-specific intermittent training can effectively enhance SJFT and CMJ performance in judo athletes, ultimately improving competition performance.

Considering the specific training principles required to elicit targeted adaptations for enhancing sport-related physical fitness and competitive performance, the regimen must be effectively designed to meet these demands (Baechle & Earle, 2008). Specific to the characteristics of combat sports, especially in simulated matches, it is intermittent in nature (Slimani et al., 2017). However, due to the short rest intervals in simulated matches, the physiological response may not exhibit the same effect. Therefore, it is necessary to implement sport-specific intermittent training to enhance athletic performance (Hernández-García, Torres-Luque & Villaverde-Gutierrez, 2009; Vigh-Larsen et al., 2024). Kamandulis et al. (2018) investigated the effects of a four-week sport-specific sprint interval training on upper-body performance. After training, it was found that upper-body punching force increased. Franchini et al., (2017) employed uchikomi as exercises in a 4-week intermittent training program, and the results showed a significant reduction in the SJFT index. This suggests that designing intermittent training that aligns with the characteristics of combat sports competitions is more beneficial for specific performance. However, their study did not assess lower-limb impulse performance or the role of sparring partners. Our study fills this gap by examining CMJ performance and the physical adaptations of trainers in judo-specific intermittent training.

In Taiwan, only the top-performing first-grade collegiate judo athletes are chosen by coaches to compete in official matches, while other athletes are assigned the role of sparring partners. These sparring partners practice ukemi (falling techniques) repeatedly during training. Ukemi is a key skill in judo that helps prevent injuries from falls and includes various types, such as mae-ukemi, ushiro-ukemi, yoko-ukemi, and forward rotating ukemi (Odaka et al., 2023). However, the impact of practicing ukemi on athletes’ overall athletic performance has not been fully established. Understanding these effects could help coaches create more targeted and effective training programs for the upcoming season.

In summary, this study aims to address two topics based on past reports. First, in addition to regular training, this study designed a six-week judo-specific intermittent training program to examine whether the intervention (uchikomi + throw (ippon seoi-nage)) could enhance the lower-limb impulse and specific performance ability of judo athletes. Second, we expect to observe the impact of sparring partners’ involvement in training, specifically by performing mae-ukemi, on judo athletes’ lower-limb impulse and specific performance ability in this study. We hypothesized that additional sport-specific intermittent training would improve sport-specific performance and lower-body impulse, while sparring partners might experience opposite effects.

## Methods

### Participants

A total of 30 healthy male adult judo athlete (all black belt) volunteers participated in this study. G*Power 3.1.9.7 was used to calculate the appropriate sample size for this study. Using a power of 0.80, an *α* of 0.05, and an total effect size of 0.30, the required total sample size was determined to be 30, which met the power criterion (Faul et al., 2007). In the assignment of groups, it is divided into the Tori group (169.90 ± 6.17 cm in stature, 76.67 ± 18.2 kg in mass, 20.64 ± 3.07 years of age and 13.10 ± 2.88 years of experience) and the Uke training group (172.36 ± 6.32 cm in stature, 77.28 ± 19.4 kg in mass, 21.73 ± 6.15 years of age and 11.90 ± 1.79 years of experience) and control group (CON) (167.18 ± 4.16 cm in stature, 76.77 ± 13.7 kg in mass, 21.55 ± 5.30 years of age, and 12.00 ± 2.40 years of experience), 10 people in each group.

### Study design

Participants were excluded if they had any contraindications to exercise, were taking drugs that altered metabolic rate, or had any cardiovascular abnormalities identified by their physicians that might have prevented them from completing the test procedure safely. Participants were required to visit the laboratory twice for pre-test and post-test sessions. They were asked to wear experimental equipment and complete approximately one hour of training (including equipment setup) in a laboratory environment with room temperature set at an average of 23 °C. If the test or training procedure failed (*e.g.*, due to training-related injury or any other factor), the participant’s profile and data were excluded from the analysis. Table 1 lists the anthropometric and body composition characteristics of the participants. This study was conducted in accordance with the principles of the Declaration of Helsinki. The study protocol, including the procedures and written informed consent form, was reviewed and approved by the Institutional Review Board of Fu Jen Catholic University (Approval Number: C110198). Written informed consent was obtained from all participants prior to their inclusion in the study.

**Table 1:** Training routine for three groups over six weeks.

	Mon.	Tue.	Wed.
Training program	Warm up Sprint (30 m*8, 50 m*6, 100 m*4) Back squat 3* 10 reps Bench press 3* 10 reps Bent over row 3* 10 reps RDL 3* 10 reps Overhead press 3* 10 reps Pull up 3* 10 reps	Warm up Tumble 20 min Uchikomi 8 min Throw* 10 reps Randori 4 min* 12 sets	Warm up Tumble 20 min Ne-Waza Uchikomi 10 min Ne-Waza Randori 3 min*6 sets Uchikomi 15 min Randori 4 min*8 sets Rope climbing traning* 8 sets
	Thu.	Fri.	
Training program	Warm up Tumble 20 min Bear craw*3 * 20 min Push up* 150 reps Uchikomi 15min	Warm up Back squat 3* 10 reps Bench press 3* 10 reps Bent over row 3* 10 reps RDL 3* 10 reps Overhead press 3* 10 reps Pull up 3* 10 reps Ne-Waza Randori 3 min* 6 set Uchikomi 15 min Randori 4 min* 8 set

**Notes.**

HIIT training, two blocks six sets and 20 s of each set, rest interval of 20 s; Training program, judo training routine of three groups; RDL, romanian deadlift; Resistance training intensity: 10 RM.

### Intermittent training procedures

Before receiving training intervention, all participants received pre-test for CMJ, SJFT, grip and body composition. A 6-weeks intermittent training program was applied to the Tori and Uke group, and the post-test was conducted after all training programs were completed. The intermittent training in this study was conducted twice per week (Tuesday and Thursday), with a 24-hour interval between sessions. Participants were paired based on a body weight difference of no more than 10%. Training sessions took place at 2:00 PM (14:00) on Tuesdays and Thursdays. In each session, the Tori group performed uchikomi + ippon seoi-nage (UI), while the Uke group assisted by executing mae-ukemi (MU). The training consisted of two blocks, each containing six sets, with each set lasting 20 s, followed by a 20-second passive rest between sets. The total training duration was 8 min. During the study period, they adhered to a consistent training model and load as prescribed in this protocol, alongside their usual judo training routines. Table 1 presents the habitual training schedule of the three groups of participants.

### SJFT procedures

The test, originally developed by Sterkowicz (1995), demonstrates good test-retest reliability with an SJFT index reliability of *α* = 0.807 and a measurement error of 4.5%, indicating its stability in assessing judo-specific performance (Štefanovský et al., 2021). The weight of two Uke members is close to that of Tori, the difference is within 10%. During the SJFT, Tori stood in the middle and two Ukes stood three m from Tori on either side. After the researchers gave the signal representing the start, Tori had to run to one of the Ukes to perform ippon seoi-nage throw, and then run to the other Uke to throw him/her. Tori must get as many numbers of throws as possible and execute at the fastest speed during the process. SJFT is mainly divided into three periods, A = 15 s; B = 30 s; C = 30 s, with 10-second intervals between them. The chest strap Polar H10 Heart Rate Monitor (Polar Electro Oy, Finland) was used to collect the heart rate at the end of SJFT (HR_post_) and 1 min after the test (HR_post1min_). The collected number of throws and HR were used to calculate SJFI. The calculation formula is: SJFI = (Final HR (bpm) + HR1 min (bpm))/Throws (N).

### CMJ test

The participants were asked to be on the force plate (9260AA; Kistler Ltd, Switzerland), and the system recorded the duration and force value during the CMJ process with a sampling frequency of 1,000 HZ. The MATLAB computer language was used to calculate key CMJ parameters, including jump height (JH), relative peak force (RFP), time to take-off (TTT), modified reactive strength index (RSImod), and rate of force development (RFD). JH was calculated from the flight time during the jump using free-fall equations. RFP was defined as the peak force generated before take-off, while RSImod was determined by dividing JH by the time to take-off (TTT). RFD was computed as the change in force (ΔF) over the change in time (Δt), representing the rate at which force is developed during the movement, and the parameters are standardized by the body weight of the participants. The recovery period between each CMJ trial was one minute. Before testing, participants completed a brief warm-up (∼10 min), which included dynamic stretching and sub-maximal jumping. The familiarization jumps consisted of five sets of single-effort CMJs and two sets of five repeated CMJs to ensure movement consistency and reduce injury risk (McMahon, Jones & Comfort, 2022; Badby et al., 2022). Participants performed three maximum-effort CMJs on the force plate. They were asked to keep their hands on their waists, and to jump as high as possible. They also must be kept in the middle of the force plate when landing, if they exceed the force plate, the score will not be counted (Guess et al., 2020; Ho et al., 2016).

### Body composition and grip strength

Use InBody^®^ 570 Body Composition Analyzer (Biospace, Inc. Seoul, Korea) was used to measure height, weight and body composition, such as percent body fat (PBF), skeletal muscle ass (SMM) and body mass (BM). Participants were required to wear light sportswear and remove metal ornaments on their bodies before the measurement to reduce the error value. Grip strength data was collected using the Takei 5401 handgrip digital dynamometer (Takei Scientific Instruments Co., Ltd, Tokyo, Japan) (Chang et al., 2021). The handgrip strength assessment was conducted following a standardized protocol to ensure consistency and reliability. Participants performed the test using their dominant hand, which was determined based on their self-reported hand preference for writing and throwing. Before testing, the dynamometer was adjusted to fit each participant’s hand size to ensure an optimal grip span. During the test, participants stood in a natural position with their shoulders adducted, holding the dynamometer without body contact, while wrist flexion was not allowed to maintain standardized testing conditions. Each participant performed three trials, with the best result recorded for analysis. The grip test required participants to perform a maximal voluntary contraction for 5 s in each trial. The test was conducted exclusively with the dominant hand, and in this study, all participants were right-hand dominant (Cortell-Tormo et al., 2013).

### Statistical analysis

All statistical analyses were conducted using SPSS (IBM Corp., New York, NY, USA). A two-way repeated measures analysis of variance (ANOVA) was employed to evaluate the effects of time effect on body composition, SJFT, and CMJ. The assumption of sphericity was examined using Mauchly’s test. If the assumption was violated, the Greenhouse-Geisser correction was applied. Bonferroni-adjusted *post hoc* comparisons were conducted only for parameters with a significant group × time interaction effect to examine simple main effects. Effect sizes for the group × time interaction were calculated using partial eta squared (*η*^2^) and interpreted as small (0.01), medium (0.06), and large (0.14). Percentage change (△%) was calculated to quantify the relative improvement in performance between pre- and post-tests. Statistical significance was set at *p* < 0.05, and data were reported as mean ± standard deviation (SD).

## Results

### Body composition and grip strength

Table 2 shows the results of this study, indicating that there were no significant differences in BM, PBF, SMM, and grip strength across all groups before and after training, nor were there significant interaction effects between time and group (*p* > 0.05). This suggests that during the training period in this study, neither body composition nor grip strength performance was significantly affected.

**Table 2:** Training routine for three groups over six weeks.

	Group (*n* = 10)	Pre (Mean ± SD)	Post (Mean ± SD)	△%	*F*	Time effect *p*	Time × group *p*	*η* ^2^
BM (kg)	Tori	76.46 ± 18.64	75.97 ± 19.04	9.68	0.14	0.98	0.98	0.00
	Uke	78.82 ± 20.42	78.21 ± 20.21	0.00				
	CON	75.78 ± 14.14	77.12 ± 14.18	1.69				
PBF (%)	Tori	19.51 ± 8.16	18.87 ± 8.12	−3.28	0.03	0.93	0.96	0.00
	Uke	17.47 ± 8.88	17.97 ± 8.46	2.86				
	CON	22.14 ± 8.79	22.77 ± 8.85	2.85				
Grip (kg)	Tori	43.83 ± 7.34	46.72 ± 9.71	−6.18	0.74	0.06	0.48	0.05
	Uke	50.93 ± 5.55	50.18 ± 6.64	−1.47				
	CON	50.18 ± 11.23	44.56 ± 10.70	−11.20				
SMM (kg)	Tori	34.53 ± 6.60	34.66 ± 6.80	0.38	0.01	0.95	0.98	0.00
	Uke	34.54 ± 1.52	35.86 ± 5.26	3.82				
	CON	33.23 ± 5.02	33.53 ± 5.00	0.90				

**Notes.**

BM, body mass; PBF, percent body fat; SMM, skeletal muscle Mass; △%, percentage difference in the mean between the pre-test and the post-test.

### Lower-limb impulse

Table 3 shows the results of this study, indicating that most CMJ variables exhibited significant improvement over time. No parameters reached a significant interaction effect, suggesting that the three training methods had consistent effects on CMJ performance in judo athletes. However, △% analysis revealed that the Tori group showed a greater improvement trend compared to the other two groups. The time × group interaction for RFD was not significant (*F* = 3.46, *p* = 0.31, *η*^2^ = 0.08), indicating similar trends among groups. Additionally, the time effect was not significant (*p* = 0.07); however, △% (12.85%) analysis showed that the Tori group exhibited a higher improvement. For RFP, the time × group interaction was not significant (*F* = 0.23, *p* = 0.70, *η*^2^ = 0.02), suggesting that the training effects followed a similar trend across all groups. However, a significant time effect was observed (*p* = 0.03), with the Tori group showing a higher △% (9.37%) improvement. For JH, the time × group interaction was not significant (*F* = 1.59, *p* = 0.22, *η*^2^ = 0.10), indicating similar trends among groups. However, a significant time effect was observed (*p* < 0.01), and △% analysis showed that the Uke group (14.49%) exhibited a greater improvement. Additionally, TTT did not show a significant interaction effect (*F* = 1.18, *p* = 0.32, *η*^2^ = 0.08) or time effect (*p* = 0.38). The time × group interaction for RSImod was not significant (*F* = 1.68, *p* = 0.20, *η*^2^ = 0.11), indicating similar trends among groups. However, a significant time effect was observed (*p* < 0.01), and △% analysis showed that the Uke group (18.29%) exhibited the greatest improvement.

**Table 3:** Training routine for three groups over six weeks.

	Group (*n* = 10)	Pre (Mean ± SD)	Post (Mean ± SD)	△%	*F*	Time effect *p*	Time × group *p*	*η* ^2^
RFD (N/kg/t)	Tori	6.43 ± 1.18	7.26 ± 1.10	12.85	3.46	0.07	0.31	0.08
	Uke	6.14 ± 1.66	6.50 ± 1.75	5.83				
	CON	6.63 ± 1.98	6.64 ± 2.26	0.17				
RFP (N/kg)	Tori	14.07 ± 1.81^#^	15.39 ± 1.85	9.34	0.23	0.03^*^	0.70	0.02
	Uke	13.64 ± 1.72	14.66 ± 2.12	7.55				
	CON	14.07 ± 2.34	14.74 ± 3.20	4.81				
JH (m)	Tori	0.38 ± 0.06	0.40 ± 0.05	5.59	1.59	0.00^**^	0.22	0.10
	Uke	0.40 ± 0.05^#^	0.40 ± 0.06	14.49				
	CON	0.35 ± 0.07	0.37 ± 0.10	5.10				
TTT (s)	Tori	0.67 ± 0.03	0.64 ± 0.04	−4.30	1.18	0.38	0.32	0.08
	Uke	0.70 ± 0.04	0.68 ± 0.06	−2.15				
	CON	0.70 ± 0.09	0.71 ± 0.06	1.86				
RSImod (ratio)	Tori	0.56 ± 0.11^#^	0.62 ± 0.08	10.14	1.68	0.00^**^	0.20	0.11
	Uke	0.50 ± 0.12^#^	0.60 ± 0.12	18.29				
	CON	0.51 ± 0.10	0.52 ± 0.14	7.21				

**Notes.**

RFD rate for development RFP relative force peak JH relative force peak TTT time to take of RSImod reactive strength index-modified

* *p* < 0.05.

** *p* < 0.01.

# Different from post-training (*p* < 0.05).

△%, percentage difference in the mean between the pre- test and the post-test.

### SJFT

The time × group interaction for SJFT total was significant (*F* = 5.44; *p* ≤ 0.01; *η*^2^ = 0.28), and a significant time effect was also observed (*p* < 0.01), indicating notable improvements in performance. *Post hoc* comparisons revealed that the Tori group demonstrated a significantly greater pre-to-post improvement compared to the other two groups (Tori: *p* < 0.01; Uke: *p* = 0.58; CON: *p* = 0.78). Regarding heart rate response, post-exercise heart rate significantly decreased after training (*p* < 0.01), indicating an improvement in physical fitness adaptation. However, no significant interaction effect was found between time and group, suggesting that the trends in heart rate reduction were similar across the Tori, Uke, and CON groups. However, △% analysis showed that both the Tori (HR_post_ = −3.34%; HR_post1min_ = −9.67) and Uke (HR_post_ −3.52%; HR_post1min_ = −6.53) groups exhibited greater reductions in heart rate compared to the CON (HR_post_ = −1.54%; HR_post1min_ = −3.25) group. In terms of SJFI, the time × group interaction was significant (*F* = 6.40, *p* < 0.01, *η*^2^ = 0.32), and a significant time effect was also observed (*p* < 0.01), indicating overall improvements in performance. *Post hoc* comparisons revealed that training effects varied among groups (Tori: *p* < 0.01; Uke: *p* < 0.01; CON: *p* = 0.18), as detailed in Table 4.

**Table 4:** Two-way repeated measures analysis of SJFT.

	Group (*n* = 10)	Pre (Mean ± SD)	Post (Mean ± SD)	△%	*F*	Time effect *p*	Time × group *p*	*Post hoc* *p*	*η* ^2^
SJFT series A	Tori	6.20 ± 0.63	6.80 ± 0.42	9.68	1.44	0.14	0.25		0.09
	Uke	5.90 ± 0.57	5.90 ± 0.57	0.00					
	CON	5.90 ± 0.57	6.00 ± 1.15	1.69					
SJFT series B	Tori	11.00 ± 1.05	12.00 ± 1.05	9.68	4.61	0.08	0.01^*^	0.00^**^	0.25
	Uke	11.10 ± 1.66	11.20 ± 1.40	0.00				0.68	
	CON	10.80 ± 0.92	10.90 ± 0.74	1.69				0.68	
SJFT series C	Tori	10.40 ± 1.51	12.00 ± 1.49	15.38	5.35	0.00^**^	0.01^*^	0.00^**^	0.28
	Uke	10.60 ± 0.84	10.90 ± 0.74	2.83				0.42	
	CON	10.60 ± 0.97	10.60 ± 0.97	0.00				1.00	
SJFT total	Tori	27.60 ± 2.76	30.80 ± 2.57	11.59	5.44	0.00^**^	0.01^*^	0.00^**^	0.28
	Uke	27.60 ± 2.50	28.00 ± 2.05	1.45				0.58	
	CON	27.30 ± 1.83	27.50 ± 1.84	0.73				0.78	
HR_post_	Tori	173.80 ± 6.05^#^	168.00 ± 7.13	−3.34	0.81	0.00^**^	0.45		0.05
	Uke	176.10 ± 9.02^#^	169.90 ± 6.92	−3.52					
	CON	181.60 ± 4.90	178.80 ± 1.81	−1.54					
HR_post1min_	Tori	152.00 ± 11.3^#^	138.6 ± 15.44	−9.67	0.86	0.00^**^	0.43		0.06
	Uke	156.30 ± 11.07^#^	146.10 ± 11.63	−6.53					
	CON	166.40 ± 8.51	161.00 ± 9.53	−3.25					
SJFI	Tori	11.89 ± 1.12^#^	10.05 ± 1.42	−15.48	6.40	0.00^**^	0.00^**^	0.00^**^	0.32
	Uke	12.14 ± 1.34^#^	11.35 ± 1.11	−6.52				0.01^*^	
	CON	12.80 ± 0.93	12.40 ± 0.75	−3.13				0.18	

**Notes.**

* Significant difference between pre- and post-test = *p* < 0.05.

** Significant difference between pre- and post-test = *p* < 0.01.

# Different from post-training (*p* < 0.05).

△%, percentage difference in the mean between the pre- test and the post-test.

## Discussions

The purpose of this study was to investigate whether judo-specific intermittent training can improve lower limb impulse and SJFT performance in judo athletes. Additionally, it examined whether sparring partners performing mae-ukemi would negatively impact athletic performance. The results showed that SJFT-related variables exhibited a significant time effect. In SJFT, the time × group interaction was significant, indicating that the magnitude of improvements differed across groups. *Post hoc* comparisons revealed that the Tori group demonstrated significantly greater post-test improvements in total throws and SJFI compared to the other two groups, suggesting that intermittent training effectively enhances sport-specific fitness and throwing ability. Although CMJ showed no significant interaction effect, a significant time effect was observed, and △% analysis indicated that the Tori group exhibited greater improvement. The Uke group did not show any significant declines in any variables, indicating that the training did not lead to performance deterioration. Additionally, their performance changes were not significantly different from those of the other groups. Body composition and grip strength measurements showed no significant time effects or time × group interactions, suggesting that the training had no notable impact on body composition or grip strength.

Judo athletes are usually categorized by BM, which means they often need to maintain or reduce their weight. Therefore, increasing SMM and reducing PBF are the expected training outcomes (Franchini, Brito & Artioli, 2012). After undergoing the intermittent training program designed in this study, no significant differences were found in SMM and PBF, indicating that it does not negatively impact athletes’ BM. Previous research is similar to the current findings. Kim et al. (2011) it was found that after eight weeks of sprint interval training, there was no change in body fat among judo athletes. Since the participants in this study were first-level athletes in Taiwan, they already had favorable body composition. In addition, the training duration was relatively short, and there was no nutritional intervention, so the lack of change was expected (Keating et al., 2017). Since the judo athletes maintained their regular training, which already included activities such as sprinting and weight training, all three groups exhibited only a time effect without a significant interaction effect. Additionally, as the specialized intermittent training program for judo did not include jumping movements, it did not produce distinct effects on CMJ performance compared to their regular habitual training.

The SJFT is associated with anaerobic capacity and neuromuscular performance capability, and the Tori group showed a significant increase in the number of throws compared to the other groups. The Tori group may have performed significantly more throwing repetitions than the other group during the training period, which could be the main reason for their performance improvements. These changes may be attributed to enhanced technical skills or neuromuscular adaptations. Similarly, the improvement in SJFT performance observed in the Tori group may be related to their participation in a greater amount of aerobic activity during the intervention. The increase in the total number of throws can be interpreted from a biomechanical perspective. The repetitive performance of the ippon seoi-nage features the specialty of plyometric training, which can enhance neuromechanical adaptations, increase trunk rotation angular velocity, shorten the duration of each throw, and further obtain an even greater number of throws from SJFT. Mañas-Paris, Muyor & Oliva-Lozano (2022) have proved this, where the influence of sports intervention on athletic performance has been observed in a three-week intermittent training combined with the ippon seoi-nage, using inertial and physiological sensors. The findings reveal an obvious decrease in SJFI. The Tori group’s significantly higher number of throws compared to the other groups may also be related to the recruitment of motor units. Regarding intermittent training, it requires rapid muscle contractions, which facilitate the recruitment of more Type II fibers and enhance the central nervous system’s ability to activate and recruit motor units. Since Type II fibers can generate greater force, when the Tori bears the weight of the Uke while performing ippon seoi-nage, it further stimulates the growth and adaptation of these muscle fibers (Atakan et al., 2021; Harridge et al., 1996). Compared to the other groups, since they did not undergo sport-specific intermittent training, their improvement in the number of throws was not superior to that of the Tori group. However, since the Uke and CON groups continued their regular team training, there was no negative impact on the specialized performance of the participants in these two groups.

All three groups exhibited a significant time effect in heart rate performance, with no significant interaction effect. This finding may be related to the physiological adaptations induced by intermittent training, which can effectively increase cardiac output and improve vascular elasticity, thereby facilitating better oxygen transport and utilization. As a result, overall cardiorespiratory endurance is enhanced (Hellsten & Nyberg, 2016). In addition, aerobic training has been shown to enhance recovery capacity by improving autonomic regulation, increasing parasympathetic reactivation, and promoting efficient cardiovascular and metabolic adaptations following exercise (Ostojic et al., 2010; Patel et al., 2017). The Uke and CON groups’ habitual training already included sprint interval training, therefore, all participants’ heart rate performance also significantly improved over time. Compared to previous studies, we extended the specialized intermittent training cycle by two weeks. Based on the percentage change between pre- and post-tests (△%), we observed that the improvements in SJFT parameters exceeded those reported by Franchini et al. (2017), including the number of throws in the SJFT, HR after the SJFT, HR 1 min after the SJFT, and the SJFT index. This suggests that a six-week specialized intermittent training program can lead to greater improvements in sport-specific performance for judo athletes.

This study demonstrated that a six-week judo-specific intermittent training program effectively improved sport-specific performance, particularly in SJFT-related parameters. The Tori group exhibited significantly greater improvements in total throws and SJFI compared to the other groups, suggesting that sport-specific intermittent training enhances neuromuscular adaptation and throwing efficiency. Although CMJ performance showed only a time effect with no significant interaction, the percentage change (△%) analysis indicated that the Tori group exhibited the greatest improvement. Additionally, no significant declines were observed in the Uke group, indicating that performing mae-ukemi during training did not negatively impact performance. Body composition and grip strength remained unchanged, suggesting that the training program did not influence these factors, likely due to the athletes’ pre-existing favorable body composition, short intervention duration, and lack of nutritional control. All groups exhibited significant time effects in heart rate performance, reflecting the positive impact of intermittent training and habitual training on cardiovascular endurance and lactate clearance. Compared to previous studies, our extended six-week intermittent training cycle led to greater improvements in SJFT performance. In summary, the results of this study are promising and applicable to first-level collegiate male judo athletes. We hope that the findings will assist sports science personnel or coaches in optimizing their training schedules.

## Limitations

This study has some limitations. The participants were first-level male judo athletes in Taiwan, so the applicability of the findings to other age groups remains uncertain. Additionally, female athletes were not included in the scope of this study. Therefore, future research should consider extending the investigation to different genders and age groups. Finally, this study focused specifically on ippon seoi-nage, and the training benefits of other judo techniques remain unclear. Further in-depth studies on other techniques are recommended for future research.

## Practical applications

The findings suggest that incorporating judo-specific intermittent training can significantly enhance SJFT, making it an effective training approach for judokas aiming to improve competition endurance. While lower-limb impulse performance (CMJ) improved over time, the lack of interaction effects suggests that intermittent training may not be sufficient to influence lower-limb impulse adaptations. Additionally, serving as sparring partners (Uke group) did not negatively impact physical performance, indicating that this role can be safely integrated into training programs without hindering athletic development. Coaches and practitioners can apply these findings to optimize training strategies by combining intermittent training with a comprehensive strength and conditioning program.

## Supplemental Information

10.7717/peerj.19491/supp-1 Supplemental Information 1 Raw data

## Additional Information and Declarations

### Data Availability

The following information was supplied regarding data availability: The raw data is available in the Supplemental File.
